# Effectiveness of early intervention programs for parents of preterm infants: a meta-review of systematic reviews

**DOI:** 10.1186/s12887-018-1205-9

**Published:** 2018-07-09

**Authors:** Shuby Puthussery, Muhammad Chutiyami, Pei-Ching Tseng, Lesley Kilby, Jogesh Kapadia

**Affiliations:** 1 Maternal and Child Health Research Centre, Institute for Health Research, University of Bedfordshire, Putteridge Bury, Hitchin Road, Luton, Bedfordshire, LU2 8LE UK; 2 Neonatal Unit, Luton and Dunstable Hospital, Lewsey Rd, Luton, LU4 0DZ UK

**Keywords:** Preterm infants, Early intervention programs, Parents, Meta-review, Neonatal health

## Abstract

**Background:**

Various intervention programs exist for parents of preterm babies and some systematic reviews (SRs) have synthesised the evidence of their effectiveness. These reviews are, however, limited to specific interventions, components, or outcomes, and a comprehensive evidence base is lacking. The aim of this meta-review was to appraise and meta-synthesise the evidence from existing SRs to provide a comprehensive evidence base on the effectiveness of interventions for parents of preterm infants on parental and infant outcomes.

**Methods:**

We conducted a comprehensive search of the following databases to identify relevant SRs: Cochrane library, Web of science, EMBASE, CINAHL, British Nursing Index, PsycINFO, Medline, ScienceDirect, Scopus, IBSS, DOAJ, ERIC, EPPI-Centre, PROSPERO, WHO Library. Additional searches were conducted using authors’ institutional libraries, Google Scholar, and the reference lists of identified reviews. Identified articles were screened in two stages against an inclusion criteria with titles and abstracts screened first followed by full-text screening. Selected SRs were appraised using the AMSTAR tool. Extracted data using a predesigned tool were synthesised narratively examining the direction of impact on outcomes.

**Results:**

We found 11 SRs eligible for inclusion that synthesised a total of 343 quantitative primary studies. The average quality of the SRs was ‘medium’. Thirty four interventions were reported across the SRs with considerable heterogeneity in the structural framework and the targeted outcomes that included maternal-infant dyadic, maternal/parental, and infant outcomes. Among all interventions, Kangaroo Care (KC) showed the most frequent positive impact across outcomes (*n* = 19) followed by Mother Infant Transaction Program (MITP) (*n* = 14). Other interventions with most consistent positive impact on infant outcomes were Modified-Mother Infant Transaction Program (M-MITP) (*n* = 6), Infant Health and Development Program (IHDP) (*n* = 5) and Creating Opportunities for Parent Empowerment (COPE) (n = 5). Overall, interventions with both home and facility based components showed the most frequent positive impact across outcomes.

**Conclusions:**

Neonatal care policy and planning for preterm babies should consider the implementation of interventions with most positive impact on outcomes. The heterogeneity in interventions and outcomes calls for the development and implementation of an integrated program for parents of preterm infants with a clearly defined global set of parental and infant outcomes.

## Background

Preterm birth, defined as birth at less than 37 completed weeks of gestation, remains a significant cause of infant mortality and morbidity worldwide. Preterm births are on the increase globally with about 15 million babies born preterm annually [1]. Compared to babies born at term, preterm babies carry a higher risk of developmental delays and learning disabilities and are increasingly vulnerable to conditions such as cerebral palsy, respiratory illnesses, feeding difficulties, and vision problems [1–6].

Caring for a preterm baby can be challenging and stressful to parents. Studies have consistently documented higher levels of stress and parenting difficulties among parents of preterm babies compared to those of babies born at term [7–15]. Parents are central to children’s health and development and successful parenting is a key element in promoting overall parental wellbeing as well as children’s physical and psychosocial development. The importance of supporting parents in the early years of their children’s lives is reflected in a range of parenting programs developed over the years [16]. There is good quality evidence to demonstrate the effectiveness of early interventions in facilitating effective parenting and thereby promoting children’s health and psychosocial development [17–20].

Various early intervention programs have been developed and delivered for parents of preterm babies and some systematic reviews (SRs) have synthesised the evidence on the effectiveness of these programmes [21–24]. While individual reviews have been successful in identifying the components and assessing the effectiveness of certain interventions on parental and infant outcomes, they often focus on specific interventions [21], components [25], or outcomes [26], which limit their ability to provide a comprehensive picture of the effectiveness of early intervention programs for the parents of preterm babies.

The aim of this review of SRs, referred to as meta-review, was to appraise and meta-synthesise the evidence from SRs to provide a comprehensive evidence base on the effectiveness of interventions for parents of preterm infants on various parental and infant outcomes.

## Methods

We followed the Preferred Reporting Items for Systematic Reviews and Meta Analyses (PRISMA) guidelines [27] for this meta-review. The review question was framed using Population, Intervention, Comparator, Outcome and Study design (PICOS) framework. The population comprised of parents of preterm babies. The interventions comprised of interventions aimed at supporting parents of preterm babies. The outcome measures were indicators of health and/or psycho social wellbeing of parents and infants. SRs were included if they met the following criteria: searched at least two electronic databases; included a method of describing how the studies were included and/or excluded; synthesised findings from individual primary studies on the effectiveness of interventions for parents of preterm babies; and have drawn conclusions on at least one parental or infant outcome. No restrictions on language or the year of publication was applied as part of the inclusion criteria. The protocol was reviewed and agreed by the members of the team.

We conducted a comprehensive systematic search of the following databases to identify all existing SRs: Cochrane library, Web of science, EMBASE, CINAHL, British Nursing Index, PsycINFO, PubMed/Medline, ScienceDirect, Scopus, IBSS, DOAJ, ERIC, EPPI centre, PROSPERO, and the electronic libraries of the authors’ institutions. Additional sources searched included Google Scholar, WHO Library, and the reference list of identified reviews. The key search terms used included [parent* OR famil* OR mother* OR father* OR preterm OR prematur* OR preterm birth OR preterm infant* OR premature infant*] AND [Intervention* OR initiative* OR process* OR program* OR effect* OR implication* OR scheme* OR strategy* OR outcome* OR educat* OR impact OR evaluat* OR support* OR delivery* OR implement*] AND [“systematic review” OR “SLR” OR “SR” OR meta-analysis* OR meta-review* OR meta- regression* OR meta-synthesis* OR “realistic review” OR “descriptive review” OR “research review” OR “thematic review” OR “explanatory review” OR “narrative review” OR “integrative review” OR “mixed method review” OR “qualitative review” OR “quantitative review” OR “research synthesis” OR “evaluation review” OR “evidence mapping” OR “evidence map review” OR “impact review” OR overview OR “evidence synthesis” OR “narrative synthesis”]. The main search was conducted between 1 February – 31 March 2016 and a subsequent updated search was conducted in August 2017. We registered ourselves on key databases such as PUBMED, Cochrane library and CINAHL to receive alerts on the publication of new articles. Identified SRs were screened by two researchers (SP and MC) using a two stage process. The first stage involved screening of all titles and abstracts based on the inclusion and exclusion criteria. Full text articles of all the included SRs in stage 1 were retrieved and screened for eligibility in stage 2.

### Methodological quality assessment and data analysis

All the included SRs were assessed for methodological quality using the Assessing the Methodological Quality of Systematic Reviews (AMSTAR) tool [28]. Both the second (MC) and third (PcT) authors independently rated the methodological quality of all the SRs. Any discrepancies in scores were examined by the first author (SP) to make the final decision. SRs were assessed on eleven items on AMSTAR with the scores for individual items summed up. A total score of 11 represented an SR of the highest quality. The scores were grouped into three equal categories by the review team: score of 8–11 represented ‘high’ quality; score of 4–7 represented ‘medium’ quality; and a score of 0–3 represented ‘low’ quality.

The data from individual SRs were extracted using a predesigned review specific tool. The tool included details on the population and interventions (components, mode & place of delivery, duration); the numerical or narrative summary findings on outcomes; and the recommendations and implications for policy and practice outlined in the SRs. Author statements about the quality of the included studies to draw conclusions, their concerns, whether they agreed with the findings, and the recommendations were also recorded.

The extracted data were synthesised narratively in line with the review objective. This involved a detailed examination of the numerical and narrative summary findings and conclusions with respect to the effectiveness on outcomes and the categorisation of effectiveness as ‘positive impact’, ‘no impact’ and ‘inconclusive’ taking into account, wherever possible, the statistical significance, and the design and quality of the included studies as reported in the SR. Meta-analysis was deemed inappropriate for this review as this was a review of SRs and meta-analysis was already conducted in some of the included SRs [29]. The outcomes were classified into three categories: mother-infant dyadic outcomes; maternal/parental outcomes; and infant outcomes.

## Results

### Study selection

**Fig. 1:**
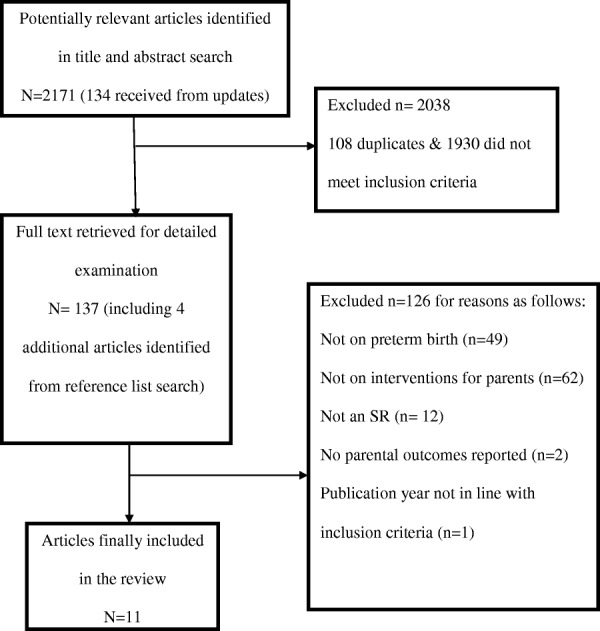
Flowchart of the SR selection process

The results of the search and SR selection are shown in Fig. 1. The initial keyword search and updates from registered databases produced a total of 2171 titles and abstracts, of which 2038 were excluded due to either discordance with the inclusion criteria or duplication from multiple databases. Full texts of the remaining 133 articles were retrieved. Four more full text articles were retrieved following reference list searches. Altogether 137 full text articles were screened against the inclusion criteria. Following full text screening, 126 articles were further excluded due to discordance with the inclusion criteria resulting in 11 SRs eligible for inclusion in the meta-review (Table 1).

**Table 1:** Characteristics of the included reviews

Authors and year of publication	Title of the study	Aim	Study designs	Included databases	Number. of studies included
Evans et al., 2014 [26]	Are parenting interventions effective in improving the relationship between mothers and their preterm infants?	To systematically review the efficacy of parenting interventions in improving the quality of the relationship between mothers and preterm infants	RCTs and quasi-experimental designs	The Cochrane Library, PubMed, CINAHL, PsycINFO and Web of Science	17
Benzies et al., 2013 [30]	Key components of early intervention programs for preterm infants and their parents: a systematic review and meta-analysis	To categorise the key components of early intervention programs and determine the direct effects of components on parents, as well as their preterm infants	RCTs	MEDLINE, EMBASE, CINAHL, ERIC, and Cochrane Database of Systematic Reviews	18
Brett et al., 2011 [25]	A systematic mapping review of effective interventions for communicating with, supporting and providing information to parents of preterm infants	To identify and map out effective interventions for communication with, supporting and providing information for parents of preterm infants.	RCTs, quasi-experimental and non-intervention studies	Medline, Embase, PsychINFO, the Cochrane library, CINAHL, Midwives Information and Resource Service, Health Management Information Consortium, Health Management and Information Service	72
Herd, et al., 2014 [32]	Efficacy of preventative parenting interventions for parents of preterm infants on later child behaviour: a systematic review and meta-analysis	To determine the efficacy of parenting interventions for parents of preterm infants to improve child behaviour	RCTs	PubMed, CINAHL, Scopus, PsychINFO, web of science, Cochrane library	12
Goyal et al., 2013 [33]	Home Visiting and Outcomes of Preterm Infants: A Systematic Review	To review evidence regarding home visiting and outcomes of preterm infants	RCTs and Cohort studies	Medline, CINAHL, Cochrane library, PsycINFO, EMBASE	17
Vanderveen et al., 2009 [23]	Early interventions involving parents to improve neurodevelopmental outcomes of premature infants: a meta-analysis	To determine whether interventions for infant development that involve parents, improve neurodevelopment at 12 months corrected age or older	RCTs	MEDLINE, CINAHL, PsychINFO, Cochrane library	25
Spittle al., 2015 [34]	Early developmental intervention programmes provided post hospital discharge to prevent motor and cognitive impairment in preterm infants	To compare the effectiveness of early developmental intervention programmes provided post hospital discharge to prevent motor or cognitive impairment in preterm (< 37 weeks) infants versus standard medical follow-up of preterm infants at infancy (zero to < three years), preschool age (three to < five years), school age (five to < 18 years) and adulthood (≥ 18 years)	RCTs and Quasi- RCTs	Cochrane Central Register of Controlled Trials, MEDLINE, CINAHL, PsycINFO, Embase	25
Boundy et al., 2016 [21]	Kangaroo Mother Care and Neonatal Outcomes: A Meta-analysis	To conduct a systematic review and meta-analysis estimating the association between KMC and neonatal outcomes	RCTs and observational studies	PubMed, Embase, Web of Science, Scopus, African Index Medicus (AIM), Latin American and Caribbean Health Sciences Information System (LILACS), Index Medicus for the Eastern Mediterranean Region (IMEMR), Index Medicus for the South-East Asian Region (IMSEAR), and Western Pacific Region Index Medicus (WPRIM).	124
Lawn et al., 2010 [31]	Kangaroo mother care’ to prevent neonatal deaths due to preterm birth complications	To review the evidence, and estimate the effect of KMC on neonatal mortality due to complications of preterm birth.	RCTs and observational studies	Cochrane Libraries, PubMed, LILACS, African Medicus, EMRO and all World Health Organization Regional Databases	15
McGregor et al., 2012 [35]	Enhancing parent-infant bonding using kangaroo care: a structured review	To review the literature on the effectiveness of kangaroo care with premature infants for enhancing bonding.	RCTs and observational studies	Medline, CINAHL, OTDBASE, PsycINFO, Applied Social Sciences Index and Abstracts (ASSIA), Allied and Complimentary Medicine Database (AMED), and British Nursing Index (BNI)	6
Zhang et al., 2014 [24]	Early Intervention for preterm infants and their mothers	To evaluate the efficacy of early interventions on maternal emotions, mother-infant interaction and infant development outcomes	RCTs	PubMed, CINAHL, EMBASE, PsychINFO, Cochrane library	12

### Characteristics of the included systematic reviews

A total of 343 quantitative primary studies were synthesised in the 11 SRs, of which 179 were Randomised Controlled Trials (RCTs). Meta-analysis was conducted in eight SRs [21, 23, 26, 30–34] and the remaining ones reported narrative syntheses. Four SRs included RCTs only [23, 24, 30, 32], while the rest included studies irrespective of the design. All except one SR [33] included primary studies without restriction to any specific geographical area although the reported interventions were mainly developed in countries such as the USA, UK, Australia, Germany, Japan, Italy, Netherlands, Norway and Columbia. One SR [33] was specifically focused on studies conducted in the US and Canada. All the included studies in another SR [31] were from low and middle income countries including Colombia, Ethiopia, Ecuador, Ethiopia, Indonesia, Bangladesh, India, Mexico and South Africa.

All the included SRs were critically appraised for methodological quality using AMSTAR tool. The result of the quality appraisal is presented in Table 2. The methodological quality assessment showed one SR with ‘high’ (score 8 to 11) quality, eight SRs with ‘medium’ (score 4 to 7) quality and two SRs with ‘low’ (0–3) quality. The included SRs had a mean AMSTAR score of 4.90. All the reviews met the AMSTAR criteria 3 and 6 (comprehensive literature search conducted and characteristics of included studies provided). The least met AMSTAR criteria among the reviews included criterion 1 (priori design provided), criterion 5 (list of included and excluded studies provided) and criterion 8 (use of scientific quality of the studies in formulating conclusions). The highest quality SR [34] was a Cochrane Collaboration review conducted using set guidelines.

**Table 2:** Quality assessment of the reviews using AMSTAR

Study	1	2	3	4	5	6	7	8	9	10	11	Total
Benzies et al., 2013 [30]	0	0	1	0	0	1	1	1	1	1	0	6
Boundy et al., 2016 [21]	0	1	1	1	0	1	0	0	1	1	1	7
Brett et al., 2011 [25]	0	1	1	1	0	1	0	0	0	0	1	5
Evans et al., 2014 [26]	0	0	1	0	0	1	1	0	1	0	0	4
Goyal et al., 2013 [33]	0	0	1	0	0	1	1	0	1	0	0	4
Herd, et al., 2014 [32]	0	0	1	0	0	1	1	0	1	0	1	5
Lawn et al., 2010 [31]	0	0	1	0	0	1	1	0	1	0	1	5
McGregor and Casey, 2012 [35]	0	0	1	0	0	1	0	0	0	0	0	2
Spittle et al., 2015 [34]	1	1	1	0	1	1	1	0	1	1	1	9
Vanderveen et al., 2009 [23]	0	0	1	0	0	1	0	0	1	0	0	3
Zhang et al., 2014 [24]	0	1	1	0	0	1	1	0	0	0	0	4

AMSTAR TOOL Key: 1 = Yes, 0 = No/Unclear/Not applicable. Areas assessed are numbered 1 to 11 on horizontal axis; 1-Priori design provided, 2-Duplicate selection/extraction, 3-Comprehensive literature search conducted, 4-Status of publication (i.e, grey literature) used as an inclusion criterion, 5-List of included & excluded studies provided, 6-Characteristics of included studies provided, 7-Quality of included studies assessed and documented, 8-Use of the scientific quality of the studies in formulating conclusions, 9-Use of appropriate methods to combine the findings of studies, 10-Assessment of publication bias, 11- Conflict of interest included

### Participants

Consistent with the focus of this meta-review, the participants were parents of preterm infants with or without their infants. The parents included mothers [21, 23, 26, 30, 31, 34, 35], fathers [30] or both parents [25, 32, 33], although the distinction was not clearly explicit in some SRs. One SR was focused on interventions targeted at black teenage mothers and mothers of lower socioeconomic status [23]. The participants in another SR were mainly first-time mothers [24] whereas two other SRs [26, 30] included only parents of first born infants who were preterm. Three SRs [21, 31, 33] included interventions for both preterm and low birth weight infants. The number of participants included in the SRs ranged from 1940 [26] to 5556 [32] although this information was not reported in two SRs [25, 35]. Participants identified in the reviews were broadly from low, middle, and high income countries, including USA, UK, Australia, Germany, Japan, Italy, Netherlands, Norway, Colombia, Ethiopia, Ecuador, Ethiopia, Indonesia, Bangladesh, India, Mexico, Sweden, Israel, South Africa, Zimbabwe and Mozambique.

### Interventions

A total of 34 parenting interventions were reported in the included reviews (Table 3). Most of the SRs reported the components of the interventions and the mode of delivery although none of the SRs included complete details of the interventions to enable replication. The intervention components were broadly classified into three categories: parent education consisting of aspects such as teaching, sensitisation, training or awareness creation; parent support consisting of guidance, encouragement or other forms of support; and infant support/ therapy consisting of infant care or therapy elements. Parent support and parent education was reported as a component in 23 and 21 interventions respectively whereas infant support/therapy was included as a component in 15 interventions.

**Table 3:** Characteristics of the interventions

Name of the intervention programme	Reviews reporting the intervention		Intervention components			Intervention focus		Mode of delivery		Place of delivery		Frequency/Duration	Additional details of the intervention
	Total number	Details provided	Parent education	Parent support	Infant support/therapy	Mother/Parent	Child	Individual	Group	Hospital	Community/ Home		
APIP	*N* = 4	N = 2	N = 2	*N* = 1	–	√	–	√	–	–	√	Weekly session for 2 years	Initiated from discharge
CAMS	N = 1	*N* = 1	*N* = 1	–	N = 1	√	√	NR	NR	NR	NR	Not Reported (NR)	Information was reported only on the intervention component
CBIP	N = 1	N = 1		N = 1	N = 1	√	√	√	–	√	–	5 inpatient sessions	–
COPE	N = 4	N = 3	N = 3	–	–	√	–	√	–	√	√	1–8 sessions before discharge (BD) and 1 week session after discharge (AD)	–
CP	*N* = 1	N = 1	N = 1	N = 1	–	√	–	√	–	√	√	5 sessions in the Neonatal Intensive Care Unit,1 home visit	Home visit within 4 weeks AD
DIG	N = 1	N = 1	N = 1	–	–	√	–	–	√	–	√	Once a day for period of 1 week	Initiated immediately AD
EG	N = 1	N = 1	N = 1	–	–	√	–	–	√	–	√	Once a day for period of 1 week	Initiated immediately AD
EI	N = 1	N = 1	N = 1	–	–	√	–	√	–	√	√	1NICU session, 1 home visit session	Home visit is done within first 60 weeks of adjusted infant age
GP	N = 2	N = 1	N = 1	N = 1	–	√	–	–	√	√	√	6 session	–
HBIP	N = 1	N = 1	–	N = 1	N = 1	√	√	√	–	–	√	8 neonatal clinic visits	Neonatal visit is initiated AD from hospital
H-HOPE	N = 1	N = 1	N = 1	–	N = 1	√	√	√	–	√	–	4 sessions at NICU	Sessions within 1 month adjusted infant age
IBAIP	N = 3	N = 3	N = 2	–	N = 3	√	√	√	–		√	6 to 8 home visits	Within 6 months AD
IC	N = 1	N = 1	–	N = 1	*N* = 1	√	√	√	–	–	√	8 sessions AD	Within 12-15 weeks
IDP	N = 1	N = 1	–	N = 1	N = 1	√	√	√	–	√	–	3-4weekly session in the hospital	Initiated AD
IFBI	N = 1	N = 1		N = 1	N = 1	√	√	√	–	–	√	3–17 sessions	Within 8 weeks AD
IHDP	N = 5	N = 5	N = 1	N = 2	N = 5	√	√	√	–	√	√	Weekly home visits for a year, then 1 visit/2 weeks for next 2 years	Sessions from discharge to 3 years of infant age
JIMHP	N = 1	N = 1	–	N = 1	–	√	–	√	–	–	√	5 sessions AD	Sessions at 1,3,5 and 12 months
KC	N = 8	N = 6	–	N = 5	N = 3	√	√	√	–	√	√	Up to 10 sessions AD	Frequency of hospital sessions not reported
KS	N = 1	N = 1	–	N = 1	–	√	–	NR	NR	–	√	4 times per day for 1 month	Start from term
MITP	N = 7	N = 5	N = 1	N = 3	–	√	–	√	–	√	√	1 session BD and 4 sessions AD	AD sessions within first 3 months
M-MITP	N = 3	*N* = 3	N = 3	N = 3	–	√	–	√	–	√	√	1 session BD and 4 sessions AD	AD sessions within first 3 months
NBAS	N = 1	N = 1	N = 1	–	–	√	–	NR	NR	NR	NR	NR	–
NCATS	N = 4	N = 1	N = 1	–	–	√	–	√	–	NR	NR	NR	–
NIDCAP	N = 2	N = 1	–	N = 1	N = 1	√	√	√	–	√	–	NR	–
NSTEP-P	N = 2	N = 2	N = 2	N = 2	–	√	–	√	–	–	√	9 home visits	Within 5 months AD
PBIP	N = 4	N = 2	N = 1	–	–	√	–	√	–	√	√	Weekly session BD and 6 sessions AD	Session before discharge starts at birth
PPI	N = 1	N = 1	N = 1	N = 1	–	√	–	√	√	√	√	5 sessions BD and 1 session AD	Sessions at home within 1-12 weeks AD
PI	N = 1	N = 1	–	N = 1	–	√	–	NR	NR	–	√	Once a month session for 12 months	Home or out-patient department
SG	N = 1	N = 1	N = 1	N = 1	–	√	–	–	√	–	√	Weekly sessions for 0-3 months, 2 sessions per month 3–9 months 1session per month 9-12 months	Start from term
SII	N = 1	N = 1	–	N = 1	N = 1	√	√	√	–	√	–	3 sessions BD	Last session on discharge day
SM	N = 1	N = 1	–	N = 1	N = 1	√	√	√	–	√	√	1 session BD or AD	–
SPEEDI	N = 1	N = 1	N = 1	–	N = 1	√	√	√	–	–	√	20 min sessions 5 times/week	Each family received at least 10 visits
TH	N = 1	N = 1	–	N = 1	N = 1	√	√	√	–	√	√	1 h blanket holding daily	Frequency not specified
VIBeS Plus	N = 2	N = 2	N = 2	N = 2	–	√	–	√	–	–	√	9 sessions in 12 months	Initiated from discharge

Key: *AD* After discharge, *BD* Before discharge, *NR* Not Reported. Interventions: *APIP* Avon Premature Infant Project, *CBIP* Clinic-Based Intervention programme, *COPE* Creating Opportunities for Parent Empowerment, *CP* Cues programme, *CAMS* Curriculum and Monitoring System, *DIG* Demonstration and interaction Group, *EI* Early intervention, *EG* Education group, *GP* Guided participation, *HBIP* Home Based intervention programme, *H-HOPE* Hospital to Home, *IDP* Individualised developmental plan, *IFBI* Individualized family-based intervention, *IBAIP* Infant Behavioural Assessment and Intervention Program, *IHDP* Infant Health and Development Program, *IC* Interaction Coaching, *JIMHP* Japanese Infant Mental Health Programme, *KC* Kangaroo Care, *KS* Kinesthetic stimulation, *M-MITP* Modified Mother Infant transaction programme, *MITP* Mother–Infant Transaction Program, *NBAS* Neonatal Behavioural Assessment Scale, *NIDCAP* Newborn Individualised Developmental & Assessment Programme, *NCATS* Nursing Child Assessment Teaching scale, *NSTEP-P* Nursing Systems Towards Effective Parenting-Preterm, *PBIP* Parent-Baby Interaction Programme, *PPI* Preventative Psychotherapy Intervention, *PI* Physiotherapy Intervention, *SII* Standardised Individualised Intervention, *SM* State Modulation, *SG* Support Group, *SPEEDI* Supporting Play Exploration and Early Development Intervention, *TH* Traditional Holding, *VIBeS Plus* Victorian Infant Brain Studies

The most frequently reported interventions were Kangaroo Care (KC) (*n* = 8) followed by Mother Infant Transaction Programme (MITP) (*n* = 7) and Infant Health and Development Program (IHDP) (*n* = 5). Fourteen interventions including Avon Premature Infant Project (APIP), Demonstration and interaction Group (DIG), Education group (EG), Home Based intervention programme (HBIP), Infant Behavioural Assessment and Intervention Program (IBAIP), Interaction Coaching (IC), Individualized family-based intervention (IFBI), Japanese Infant Mental Health Programme (JIMHP), Kinesthetic stimulation (KS), Nursing Systems Towards Effective Parenting-Preterm (NSTEP-P), Physiotherapy Intervention (PI), Support Group (SG), Supporting Play Exploration and Early Development Intervention (SPEEDI), Victorian Infant Brain Studies (VIBeS Plus) were home based. Facility based interventions included Clinic-Based Intervention programme (CBIP), Hospital to Home (H-HOPE), Individualised Developmental Plan (IDP), Newborn Individualised Developmental & Assessment Programme (NIDCAP), and Standardised Individualised Intervention (SII). Interventions with both home and facility based components included KC, MITP, IHDP, Creating Opportunities for Parent Empowerment (COPE), Cues programme (CP), Early intervention (EI), Guided participation (GP), Modified-Mother Infant transaction programme (M-MITP), Parent-Baby Interaction Programme (PBIP), Preventative Psychotherapy Intervention (PPI), State Modulation (SM), Traditional Holding (TH) had both home and facility based components. All of the interventions were focused on mothers/parents although programs such as CAMS, CBIP, HBIP, H-HOPE, IDP, IHDP, IFPI, IC, KC, NIDCAP, SM, SII, SPEEDI, TH, and IBAIP had components for the parents and their babies.

Most of the interventions were provided on an individual basis (*n* = 27) and were administered by a range of professionals including nurses, psychologists sociologists, community health workers, physiotherapists, educationists and graduate students. Half of the interventions (*n* = 17) were initiated soon after birth in the Neonatal Intensive Care Unit (NICU) whereas the others had components delivered before and after discharge from the hospital. The control groups reported in the SRs consisted of parents and babies who received the usual care for preterm infants or those who received conventional/standard information given to parents following the birth of a preterm baby. Two SRs reported follow up measurements for infant outcomes up to 18 years of the infant’s age [32, 34].

### Effectiveness of interventions on outcomes

#### Mother-infant dyadic outcomes

As presented in Table 4, the effectiveness of various interventions on mother-infant dyadic outcomes were reported in five SRs [24, 26, 30, 33, 35], with three reporting findings from meta-analyses [26, 30, 33]. All of these SRs reported improvements with respect to different mother-infant dyadic outcomes. In their meta-analysis, Evans et al., [26] found statistically significant improvements in the quality of the maternal-infant relationship for the intervention groups with effect sizes ranging from small, 0.38 to large, 2.81 from SM, NSTEP-P, KC, TH, and MITP. The same review [26] also found positive impact with large effect sizes for KC on the outcomes of symmetrical co-regulation (2.72) and asymmetrical co-regulation (− 2.81) and for mutual attention from MITP (1.95).

**Table 4:** Effectiveness on mother - infant dyadic outcomes

Mother- infant dyadic outcomes	Review	Intervention	Effectiveness on the outcome			Additional information on impact
			Positive impact	No impact	Inconclusive	
Quality of the mother–infant relationship	Evans et al., 2014 [26]	SM, NSTEP-P, KC, TH, MITP	√	–	–	Effect sizes ranged from small, 0.38 to large, 2.81
Symmetrical co-regulation		KC	√	–	–	large effect size 2.72
Asymmetrical co-regulation		KC	√	–	–	large effect size −2.81
Mutual attention		MITP	√	–	–	large effect size 1.95
Maternal sensitivity and/or responsiveness in interactions with the infant	Benzies et al., 2013 [30]	PBIP, COPE, MITP, M-MITP, NSTEP-P	–	–	√	Overall effect was not significant. Pooled effect Z = 1.84 (*P* = 0.07). Included studies showed positive effect of MITP and M-MITP
	Zhang et al., 2014 [24]	H-HOPE, MITP, COPE, EI	√			No effect size reported
Mother –infant attachment	McGregor et al., 2012 [35]	KC	√	–	–	Five of the six studies reported significant improvements
Mother-infant interaction	Goyal et al., 2013 [33]	Home based interventions (unspecified)	√	–	–	No effect size reported. 13 of the 14 studies reported positive intervention effect on any parent-infant interaction measures
	McGregor et al., 2012 [35]	KC	√	–	–	At 6 months, mother-infant interactions were significantly more optimal for the KC group (*p* < 0.05).
	Zhang et al., 2014 [24]	MITP, M-MITP, COPE, H-HOPE, EI	√	–	–	No effect size reported

Interventions: *COPE* Creating Opportunities for Parent Empowerment, *EI* Early intervention, *H-HOPE* Hospital to Home, *KC* Kangaroo Care, *M-MITP* Modified Mother Infant Transaction Programme, *MITP* Mother–Infant Transaction Program, *NSTEP-P* Nursing Systems Towards Effective Parenting-Preterm, *PBIP* Parent-Baby Interaction Programme, *SM* State Modulation, *TH* Traditional Holding

Positive impact on maternal sensitivity and responsiveness while interacting with the infant was reported from five interventions including H-HOPE, MITP, COPE, and EI [24] although the effect size was not available. In their meta-analysis, Benezies et al., [30] found limited impact of early intervention programs including PBIB, COPE, MITP, M-MITP, NSTEP-P on maternal sensitivity and responsiveness. The authors, however, stated that two of the included studies showed a positive impact of MITP and M-MITP [30]. McGregor et al., [35] reported significant improvements in mother-infant attachment following KC based on findings from five of the six studies included in their review. Overall improvements in mother-infant interaction were reported from MITP, M-MITP, COPE, H-HOPE, EI [24] and KC [35] and from home based interventions with active parental involvement [33].

Overall, KC and MITP showed most consistent positive impact on mother–infant dyadic outcomes. KC had positive impact on the quality of the mother-infant relationship, symmetrical co-regulation, asymmetrical co-regulation [26], mother-infant attachment [35], and mother-infant interaction [35]. MITP showed positive impact on the quality of the mother-infant relationship, mutual attention [26], maternal sensitivity and/or responsiveness [24, 30] and mother-infant interaction [24]. Most of the interventions (KC, MITP, TH, COPE, EI) with positive impact on various mother-infant dyadic outcomes had both home and facility based components [24, 26, 35]. Among interventions that are exclusively home based, NSTEP-P improved mother infant relationship (effect size 0.38) [26] but had no effect on sensitivity/responsiveness [30]. Among facility based interventions, H-HOPE showed positive impact on sensitivity/responsiveness although no effect size was indicated [24].

#### Maternal/ parental outcomes

The effectiveness of the interventions on a range of maternal/ parental outcomes was reported across the SRs as shown in Table 5. Improvement in the quality of the mother–infant relationship for mothers was reported in two of the SRs [25, 26]. In their meta-analysis of RCTs, Evans et al., [26] found significant improvements in mother – infant relationship for the mothers who took part in GP and for mothers with low education in State Modulation-Nursing System Towards Effective Parenting-Preterm (SM-NSTEP-P) based on self-report questionnaires from the mother’s perspective [26]. Parent led peer support groups in the NICU also improved mother – infant relationship for mothers of critically ill preterm babies although the reported evidence was based on a non- RCT study [25].

**Table 5:** Effectiveness on maternal/parental outcomes

Maternal/parental outcomes	Review	Intervention	Effectiveness of the intervention on outcome			Additional information on impact
			Positive impact	No impact	Inconclusive	
Quality of mother–infant relationship for mothers	Evans et al., 2014 [26]	GP	√	–	–	Large effect sizes using observation measure (2.09) and interview measure (1.20)
		SM-NSTEP-P	√	–	–	Positive impact for mothers with low education, with effect size 0.86
	Brett et al., 2011 [25]^a^	Parent support groups/parent led peer support	√	–	–	Evidence reported from a non-RCT study for mothers of critically ill preterm babies
Maternal/parental stress alleviation	Benzies et al. 2013 [30]	M-MITP, NBAS, COPE, PBIP, IBAIP	–	–	√	Pooled effect z = 0.40 (*p* = 0.69)
	Brett et al., 2011 [25]^a^	COPE, MITP, NIDCAP	√	–	–	Evidence reported from four high quality and well conducted RCTs. No significant reduction in parental stress from NIDCAP at 1–2 weeks after the baby was born
		Home support programmes where parents are visited regularly for the first year and for upto three years afterwards	√	–	–	Based on RCT evidence with high risk of bias. Specific details of the intervention unclear
	McGregor et al., 2012 [35]	KC	√	–	–	Reduction in stressful situations (32%), heart rate (7%) and Pain Visual Analogue Scale score (89%)
	Zhang et al., 2014 [24]	M-MITP, COPE, MITP	√	–	–	Impact until the baby is 12 months old with MITP
Reduction in maternal/parental anxiety	Benzies et al. 2013 [30]	COPE, VIBeS Plus, NBAS	√	–	–	Positive pooled effect z = 2.54 (*P* = 0.01)
	Zhang et al., 2014 [24]	Not specified	–	√	–	Used State Trait Anxiety Inventory scale to measure anxiety
	Brett et al., 2011 [25]^a^	KC	√	–	–	Significant reduction in maternal anxiety around her infant. RCT evidence showing music during KC resulted in significantly lower maternal anxiety
Reduction in maternal depressive symptoms	Benzies et al. 2013 [30]	COPE, VIBeS Plus, M-MITP,	√	–	–	Positive pooled effect z = 4.04(P < 0.0001)
	Zhang et al., 2014 [24]	MITP, COPE	√	–	–	Positive impact on depressive symptoms after the infant was discharged home. Statistical significance not reported
	Brett et al., 2011 [25]^a^	KC	√	–	–	Significantly less postnatal depression compared with the controls at 37 weeks
Maternal self-efficacy	Benzies et al. 2013 [30]	NBAS	√	–	–	Pooled effect z = 2.05 (P = 0.04)
Parental confidence/ competence/ satisfaction	Goyal et al., 2013 [33]	Home visiting programmes	√	–	–	Name of the interventions not specified
	Brett et al., 2011 [25]^a^	MITP, KC	√	–	–	MITP significantly improved maternal satisfaction and maternal self-confidence. KC provided the mother with a significantly greater sense of competence with their infant
		Breast feeding support programmes	√	–	–	Improved the confidence of mothers in breastfeeding
		NIDCAP	–	√	–	RCT evidence showing no impact at 1–2 weeks after birth
Mother’s/Parents’ interaction with infants	Brett et al., 2011 [25]^a^	Discharge planning programmes	√	–	–	Based on RCT evidence with high risk of bias. Specific details of the intervention unclear
		Home support programmes	√	–	–	Based on RCT evidence with high risk of bias. Specific details of the intervention unclear
		KC	√			Significantly greater sensitivity towards her infant. Effect size not reported. Better infant interaction, more touch, better adaptation to infant cues and better perception of their infant at all time periods.
Mother’s coping skills	Zhang et al., 2014 [24]	COPE	–	–	√	Both positive and no impact reported.
Preparing parents to see infant for first time	Brett et al., 2011 [25]^a^	Use of photograph	√	–	–	Reported positive effect based on a well conducted RCT
Parents’ emotional and practical guidance	Brett et al., 2011 [25]^a^	Home based support programmes	√	–	–	^a^RCT (1-), interventions unclear

^a^Brett et al., [25] used evidence from RCTs with the strength of evidence reported using Scottish Intercollegiate Grading Network guideline

Interventions: *COPE* Creating Opportunities for Parent Empowerment, *GP* Guided participation, *IBAIP* Infant Behavioural Assessment and Intervention Program, *KC* Kangaroo Care, *M-MITP* Modified Mother Infant transaction programme, *MITP* Mother–Infant Transaction Program, *NBAS* Neonatal Behavioural Assessment Scale, *NIDCAP* Newborn Individualised Developmental & Assessment Programme, *NSTEP-P* Nursing Systems Towards Effective Parenting-Preterm, *PBIP* Parent-Baby Interaction Programme, *SM* State Modulation, *VIBeS Plus* Victorian Infant Brain Studies

Reduction in maternal and/or overall parenting stress was reported in three SRs from the following interventions: M-MITP, COPE, MITP [24], COPE, MITP, NIDCAP [25] and KC [35]. Brett et al.’s [25] findings relating to MITP, COPE and NIDCAP were based on well conducted RCTs. Brett et al., [25] also indicated a recent RCT suggesting no significant reduction in parental stress from NIDCAP at 1–2 weeks after the baby was born. McGregor et al., [35] reported significant reduction in maternal stress from KC, while Zhang et al., [24] reported MITP to be effective in alleviating maternal stress up to 12 months. In their meta-analysis, Benzies et al., [30] reported inconclusive evidence on the impact of M-MITP, Neonatal Behavioural Assessment Scale (NBAS), COPE, PBIP, IBAIP on stress (z = 0.40 *p* = 0.69).

Three SRs [24, 25, 30] reported changes in maternal/parental anxiety, with one [30] reporting strong effect from COPE, NBAS and VIBeS Plus on maternal anxiety reduction based on a meta-analysis (z = 2.54 *p* = 0.01) and another [25] reporting positive effect on maternal anxiety reduction from KC. The third SR [24] found no statistically significant effect on parental anxiety reduction from early interventions in general although the interventions were not specified. One SR [30] reported reduction in maternal depressive symptoms from COPE, VIBeS Plus, and M-MITP with strong statistical effect (z = 4.04 *P* < 0.0001). Although two SRs reported impact of MITP, COPE [24] and KC [25] on reduction in maternal depressive symptoms, the statistical significance was not reported.

Benzies et al., [30] found improvements in maternal self-efficacy from NBAS with strong statistical effect [z = 2.05 (*P* = 0.04)]. Home visiting interventions in general were found to significantly improve mother’s confidence and satisfaction at 6 months postnatally [33]. MITP, KC, breast feeding support [25] and home visiting programmes [33] showed positive impact on maternal confidence and competence. NIDCAP had no significant impact on parental confidence at 1–2 weeks [25]. Discharge planning programs, home support programs and KC appeared to improve maternal/parental interaction with infants [25]. Zhang et al., [24] reported significant improvements in mother’s coping skills from COPE.

Overall, the interventions with positive impact on most parental/maternal outcomes were KC (*n* = 5), MITP (*n* = 3) and COPE (n = 3). KC had positive impact on stress alleviation [35], reduction in maternal anxiety [25], reduction in depressive symptoms [25], parental confidence/competence/satisfaction [25] and parent’s interaction with infants [25]. MITP had positive impact on stress alleviation, parental confidence/competence/satisfaction [25], and reduction in depressive symptoms [24]. COPE had positive impact on stress alleviation [24, 25], reduction in anxiety [30] and reduction in depressive symptoms [30]. Most of the interventions (KC, MITP, COPE, GP, SM-NSTEP-P, COPE, M-MITP), with positive impact on maternal/parental outcomes had both home and facility based components [24–26, 30, 35]. Few home-based interventions (NSTEP-P, SG, VIBeS Plus) showed positive impact on mother’s quality of relationship, parental confidence and reduction in anxiety/depressive symptoms [25, 26, 30, 33]. It would appear interventions that were exclusively facility-based had little impact on maternal/parental outcomes.

#### Infant outcomes

The effectiveness of interventions on a range of infant outcomes was reported across the reviews as shown in Table 6. The impact was measured using a range of tools at various ages; examples included Bayley Scales of Infant Development [23, 33, 34]; Griffiths Mental Development Scale, McCarthy Scales of Children’s Abilities, Stanford-Binet Intelligence Scale, Wechsler Preschool and Primary Scale of Intelligence [23, 34]; Differential Abilities Scale Edition II, Wechsler Intelligence Scale for Children - Full Scale IQ, Kaufman Assessment Battery for Children, British Abilities Scale, Wechsler Abbreviated Scale of Intelligence [34]; and Behaviour Assessment System for Children-Preschool version [32].

**Table 6:** Effectiveness on infant outcomes

Infant outcomes	Review	Intervention	Effectiveness of the intervention on the outcome			Additional information on impact
			Positive impact	No impact	Inconclusive	
Infant’s quality of relationship with mother	Evans et al. 2014 [26]	KC, TH, SM NSTEP-P	√	–	–	Effect sizes ranged from small, 0.35 to large, − 1.60. Large effect size observed with KC (1.60) and TH (− 0.87)
Behaviour improvement	Herd et al. 2014 [32]	IHDP, M-MITP, VIBeS Plus	√	–	–	Small, but significant, effect on behaviour outcomes. IHDP improved behaviour up to 3 years of age, the VIBeS Plus program up to 4 years and the M- MITP up to 5 years
		APIP	–	√	–	No improvement in child behaviour
	Zhang et al., 2014 [24]	MITP, COPE	√	–	–	Symbolic behaviour (understanding spoken language /object use in play)
Temperament	Benzies et al. 2013 [30]	M-MITP	√	–	–	Positive effect at 3 and 6 months. Effect size not reported
	Zhang et al., 2014 [24]	MITP, COPE	√	–	–	Statistical significance not reported
Nutrition and growth	Goyal et al., 2013 [33]	IHDP, others not specified	–	–	√	Mixed findings with one study demonstrating a significant intervention effect on weight and length during infancy (at 4 and 12 months)
	Boundy et al., 2016 [21]	KC	–	√	–	No improvements in weight gain or body length growth
Breast feeding	Boundy et al., 2016 [21]	KC	√	–	–	Improvements in exclusive breast feeding
	Zhang et al., 2014 [24]	MITP, COPE	√	–	–	Improvements in general breast feeding
Height and head circumference	McGregor et al., 2012 [35]	KC	√	–	–	Improvements in height & head circumference reported by one study
Head circumference	Boundy et al., 2016 [21]	KC	√	–	–	Improvements in head circumference
Decrease in infant heart rate and pain	Boundy et al., 2016 [21]	KC	–	√	–	Ineffective with respect to heart rate, respiration, and pain experience
	McGregor et al., 2012 [35]	KC	√	–	–	Infant’s heart rates and pain scores significantly decreased during intervention (*p* = .007 and *p* = .005, respectively) and post-intervention (*p* = .03 and *p* = .04, respectively), although there was no significant differences in infants’ stress levels
Reduction in morbidity and health service utilisation	Goyal et al., 2013 [33]	IHDP	–	–	√	Mixed findings. Small, statistically significant increase in maternally reported minor illnesses at 3 years of age, but only for infants weighing, 1500 g, and no effect on serious health conditions. No significant effects on rates of hospitalization or acute care visits
	Boundy et al., 2016 [21]	KC	√	–	–	RR = 0.53 (Neonatal sepsis), RR = 0.22 (Hypothermia), RR = 0.12 (Hypoglycemia)
	Lawn et al., 2010 [31]	KC	√	–	–	RR = 0.34 (RCT evidence)
Reduction in hospital readmission	Boundy et al., 2016 [21]	KC	√	–	–	Reduced hospital readmission by 58%
Lower mortality	Boundy et al., 2016 [21]	KC	√	–	–	Significant protective effect on mortality. Mortality 36% lower among low birth weight new borns.
	Lawn et al., 2010 [31]	KC	√	–	–	Large effect size, RR = 0.49 (RCT evidence) and RR = 0.68 (non-RCT evidence)
Early mental development/neurodevelopment	Vanderveen et al., 2009 [23]	APIP, KC, COPE, IHDP, NIDCAP, others not specified	√	–	–	Large effect size at 6 months Weighted Mean Difference (WMD) = 3.55, *p* = 0.05), 12 months (WMD = 5.57, *p* = 0.0009), 24 months (WMD = 7.59, *p* = 0.0003) and 36 months (WMD = 9.66, p < 0.0001)
	Zhang et al., 2014 [24]	MITP, COPE	√	–	–	Statistical significance not reported
Long term mental development (at 5 years)	Vanderveen et al., 20,092 [23]	APIP, IHDP, others not specified	–	√	–	WMD = −1.36, (*P* = 0.24)
Early cognitive development (infancy & preschool age)	Spittle et al., 2015 [34]	Early interventions including MITP, IHDP, M-MITP, IBAIP, CBIP, HBIP, SPEEDI, others not specified	√	–	–	Infancy -developmental quotient (DQ): standardised mean difference (SMD) 0.32 [0.16, 0.47]; *P* < 0.001; 16 studies; 2372 participants. Preschool age -intelligence quotient (IQ); SMD 0.43 [0.32–0.54]; *P* < 0.001; eight studies; 1436 participants.
	Benzies et al. 2013 [30]	M-MITP, NBAS	√	–	–	Effective at 4 months (NBAS) and 3 and 6 months (M-MITP)
Long term cognitive development	Spittle et al., 2015 [34]	MITP, IHDP, APIP	–	√	–	School age – IQ: SMD 0.18 [− 0.08, 0.43]; *P* = 0.17; five studies; 1372 participants
Early motor development	Spittle et al., 2015 [34]	Early interventions including MITP, IHDP, M-MITP, IBAIP, CBIP, HBIP, SPEEDI, others not specified	√	–	–	Small significant effect in motor development in infancy. Motor scale DQ: SMD 0.10 [0.10, 0.19]
Long term motor development			–	√	–	SMD −0.18, 95% CI -0.47 to 0.11; *P* = 0.22. Only five included studies reported outcomes at preschool age (*n* = 3) or at school age (*n* = 2).
Early psychomotor development	Vanderveen et al., 2009 [23]	IHDP, NIDCAP, others not specified	√	–	–	6 months WMD = 3.47 (_3.92, 10.86) *P* = 0.36, 12 months WMD = 5.10 (1.44, 8.75) *P* = 0.006, 24 months WMD = 2.47 (_2.01, 6.94) *P* = 0.28)
General child development	Benzies et al. 2013 [30]	VIBeS Plus	√	–	–	Short term 0–24 months
	Goyal et al., 2013 [33]	Home visiting interventions	√	–	–	Overall effect at infancy, z = 6.98 (*p* < 0.001)
	Zhang et al., 2014 [24]	M-MITP, COPE, MITP	√	–	–	Overall development up to 12 months

Interventions: *APIP* Avon Premature Infant Project, *CBIP* Clinic-Based Intervention programme, *COPE* Creating Opportunities for Parent Empowerment, *HBIP* Home Based intervention programme, *IBAIP* Infant Behavioural Assessment and Intervention Program, *IHDP* Infant Health and Development Program, *KC* Kangaroo Care, *M-MITP* Modified Mother Infant transaction programme, *MITP* Mother–Infant Transaction Program, *NBAS* Neonatal Behavioral Assessment Scale, *NIDCAP* Newborn Individualised Developmental & Assessment Programme, *NSTEP-P* Nursing Systems Towards Effective Parenting-Preterm, *SM* State Modulation, *SPEEDI* Supporting Play Exploration and Early Development Intervention, *TH* Traditional Holding, *VIBeS Plus* Victorian Infant Brain Studies

Improvement in the quality of the mother–infant relationship for infants was reported from KC, TH, SM, NSTEP– P with effect sizes ranging from small, 0.35 to large, − 1.60 [26]. Small, but significant, improvements were reported in child’s general behaviour at different ages from M-MITP (at 5 years), VIBeS Plus (at 4 years) and IHDP (at 3 years) [32]. Similarly, MITP and COPE were found to be effective towards improving symbolic behaviour of infants with respect to understanding spoken language/object use during play [24]. Benzies et al., [30] and Zhang et al., [24] found positive effect of M-MITP [24, 30] and COPE [24] on child temperament although the strength of the effect was not reported.

The impact of IHDP on physical growth and nutritional status was inconclusive [33] while KC had no clear positive impact on weight gain or body length growth [21]. Kangaroo Care had positive impact on exclusive breast feeding KC [21] while MITP and COPE resulted in improvements in general breast feeding [24]. KC was also beneficial in improving head circumference [21, 35] and height [35]. The impact of KC in reducing infant heart rate and pain was inconsistent with one SR reporting no impact [21] and another SR reporting positive impact [35].

Morbidity related outcomes were reported in three SRs [21, 31, 33]. Goyal et al., [33] found mixed impact of IHDP on reduction of morbidities with small, statistically significant increase in maternally reported minor illnesses at 3 years of age, but only for infants weighing 1500 g, and no effect on serious health conditions or on rates of hospitalization or acute care visits. KC significantly reduced relative risk (RR) of morbidities generally [21, 31], especially neonatal sepsis, hypothermia, hypoglycaemia and hospital readmission [21]. The significant protective effect of KC on infant mortality was reported in two of the SRs [21, 23] based on evidence from RCTs exclusively in one [21] and a combination of RCTs and non-RCTs in the other [31].

Positive impact of various interventions on a number of child developmental outcomes from both RCT and non-RCT studies were reported in five SRs [23, 24, 30, 33, 34]. Vanderveen et al., [23] examined child mental development outcomes including the level of cognitive, language and personal-social development at ages of 6 months, 12 months, 24 months, 36 months and 5 years, and found statistically significant impact at different ages with the impact peaking at 36 months. The impact decreased thereafter, eventually becoming insignificant at 5 years [23]. Zhang et al., [24] found MITP and COPE to be effective in promoting symbolic behaviour including understanding of spoken language and object use in play and communication. Similarly, Spittle et al., [34] examined the impact of early developmental interventions in general on cognitive and motor outcomes and found strong positive effect on cognitive development from 0 to 5 years. The effect on cognitive development was not maintained after 5 years. The same SR also found that the effect on motor development remained positive with small effect size for 0 to 2 years, but became insignificant thereafter [34]. Based on evidence from RCTs, Benzies et al., [30] found positive impact of M-MITP (3–6 months) and NBAS (4 months) on early cognitive development. Vanderveen et al., [23] found positive impact of early interventions including IHDP and NIDCAP on psychomotor development. Zhang et al., [24], Benzies et al., [30] and Goyal et al., [33] reported positive impact of MITP, M-MITP and COPE up to 12 months of infant age [24], VIBeS Plus upto 24 months [30], and home visiting interventions (age unspecified) [33] on general infant development.

Overall, KC had the most frequent positive impact on infant outcomes (*n* = 9) followed by MITP (*n* = 7), COPE (*n* = 5), M-MITP (n = 5) and IHDP (n = 5). KC had positive impact on infant’s quality of relationship with mother [26], breast feeding [21, 24], height [35], height and head circumference [21, 35], decrease in infant heart rate and pain [35], reduction in morbidity [21, 31], reduction in hospital readmission [21], lower mortality [21, 31], early mental development/ neurodevelopment [23]. Most of the interventions (KC, MITP, COPE, M-MITP, IHDP, TH, SM) that showed positive impact on various infant outcomes (infant’s quality of relationship, infant’s behaviour, breast feeding, head circumference, infant’s height, mental development, psychomotor development, early motor development, early cognitive development, general development at infancy, temperament and reduced hospital readmission/mortality had both home and facility based components [21, 23, 24, 26, 30, 32, 34, 35]. Interventions that were exclusively home based (NSTEP-P, VIBeS Plus, IBAIP, HBIP, SPEEDI) improved infant’s quality of relationship, behaviour, cognitive development, early motor development and overall development in infancy [26, 32–34]. Two facility-based interventions (CBIP, NIDCAP) were found to improve cognitive development, psychomotor development and motor development in infancy, although the effect did not sustain in later ages [23, 34].

## Discussion

This meta-review appraised and synthesised the evidence from 11 SRs on the effectiveness of early interventions on mother-infant dyadic, maternal/parental, and infant outcomes. To our knowledge, this is the first meta-review that was conducted with a specific focus on the effectiveness of interventions for parents of preterm infants on both parental and infant outcomes. Majority of the SRs were rated as of high or medium methodological quality. We found 34 interventions reported in the included SRs with differing components delivered by various professionals in the health facility and/or home settings. All the identified interventions started after the baby was born, either at the health facility or at home after discharge. Great majority of the interventions were focused on mothers whereas interventions specifically focusing on fathers or both the parents were relatively few. Although some SRs focused on interventions targeted at specific groups such as black teenage mothers and mothers of lower socioeconomic status [23], first-time mothers [24] and parents of first born infants who were preterm, we could not find any reviews specific to groups at higher risk of preterm birth, or reviews exclusively based on studies from low and middle income countries for interventions other than KC.

The most frequently reported interventions in our meta-review included the well-established programs: KC, MITP and IHDP. While KC has been defined with four key components - early, continuous, and prolonged skin-to-skin contact between the new-born and mother; exclusive breastfeeding; early discharge from the health facility; and close follow-up at home [36], there were variations in their implementation across the SRs. The theoretical foundations of MITP and IHDP have been highlighted by some SRs to demonstrate their positive impact. MITP is rooted in the transactional theory of development [37] arguing that children’s developmental outcomes are shaped by the dynamic interplay between the child’s behaviour, the caregiver’s response, and the contextual factors that may influence both the child’s behaviour and the caregiver response [38]. This framework emphasised children’s active role in a reciprocal interaction that influences their own development [37]. MITP helps to enable the parents to appreciate their infant’s unique characteristics, temperament and developmental potential, gradually sensitizing parents to infant cues, thereby improving the interaction between the parents and the infants [25]. The modified version, M-MITP was designed to support mothers of preterm infants up to 5 years of age based on the premise that mothers’ experiences of the preterm infant will transform over time and improve connection between the mother and the infant [32, 37]. The programme also encouraged engagement from both fathers and mothers, which eventually appeared to enhance their commitment to the programme. IHDP is underpinned by the wider bio-psychosocial model of early development which views the child’s social and cognitive development as influenced by the extent of parent support, cultural environment, health status and genetics [39]. The programme included both home and facility based approaches designed to enhance the cognitive, behavioural, and health status of the infant, with the parent considered as an essential participant.

The interventions with most frequent positive impact across all the outcomes were KC and MITP, with KC standing out as the programme with the most positive impact on mother–infant dyadic, maternal/parental and infant outcomes. COPE also showed effectiveness on maternal/parental and infant outcomes. COPE provided an educational programme for parents at the neonatal unit including aspects such as the appearance and behavioural characteristics of preterm infants, how parents can participate in their infant’s care, and how parents can make more positive interactions with their infant [25]. Other programs that showed consistent positive impact on infant outcomes were M-MITP and IHDP. Several outcomes such as mother-infant interaction; maternal/parental stress alleviation; reduction in maternal anxiety; depressive symptom reduction; reduction in infant morbidity and health service utilisation were reported in at least three reviews. However, the outcomes that were reported with consistent positive impact in at least three reviews were maternal/parental stress alleviation; depressive symptom reduction; and general child development.

Our meta-review provided a comprehensive evidence base on the range of interventions to support parents of preterm babies and their effectiveness on parents and preterm infants. The rigorous methodological approach based on a focused research question with a comprehensive search strategy, clear inclusion and exclusion criteria, and structured data extraction and quality assessment using standardised techniques make our findings robust and reliable. However, our findings are limited to SRs that either involve parents or reported parent outcomes and some of the inconsistent findings with respect to the effectiveness on the outcomes may be attributed to methodological factors including the variability in the definitions and measurement approaches of individual outcomes, variability in the intervention components and their delivery, and the quality of the individual studies included in the SRs. While all the reviews provided some description of the intervention components, none of the reviews reported complete details of all the interventions to enable replication. There was considerable heterogeneity in the structural framework of the interventions and the outcomes with a range of mother-infant dyadic, parental (mainly maternal), and infant outcomes making it challenging to compare and contrast the effectiveness of different interventions. There were also inconsistencies in the way individual outcomes were measured and reported both within and across the SRs. These are significant limitations of the existing SRs.

As a meta-review of SRs, our findings are limited to the direction of the association, with indications of significance wherever possible, rather than providing the magnitude of the association itself [29]. We were able to neither assess results separately by study designs nor account for any overlapping effects that might have existed due to the studies being included in more than one SR [40]. We were also unable to assess any moderating effects of the operational or contextual factors that could have impacted the effectiveness of the interventions. Although we did not restrict language of publication, we could only identify SRs published in English which might have led to the inadvertent exclusion of relevant papers published in other languages although this is likely to be minimum.

## Conclusion

Our findings offer relevant insights and directions towards planning and implementing early intervention programs for parents to improve both parental and infant wellbeing following preterm birth. While we found a large number of interventions with considerable heterogeneity in structural framework and the outcomes, some interventions were more successful than others in achieving the intended outcomes. Neonatal care policy and planning for preterm babies should consider interventions with the most positive impact on parental and infant outcomes. The heterogeneity in interventions and outcomes calls for the development and implementation of an integrated intervention program for parents of preterm infants with a clearly defined standardised set of parental and infant outcomes.

Future meta-reviews should focus on the variations in contextual and implementation factors that can moderate the effectiveness on interventions, and on summarising the evidence by study design. Individual SRs should be conducted on the impact of interventions on groups potentially at higher risk of preterm birth such as parents from ethnic minority groups and those from low socio-economic status; and on interventions exclusively from low and middle income countries.

## Abbreviations

AMSTAR: Assessing the Methodological Quality of Systematic Reviews
APIP: Avon Premature Infant Project
CBIP: Clinic Based Intervention Programme
COPE: Creating Opportunities for Parent Empowerment
EI: Early Intervention
HBIP: Home Based Intervention Programme
H-HOME: Hospital to Home
IBAIP: Infant Behavioural Assessment and Intervention Program
IHDP: Infant Health and Development Program
KC: Kangaroo Care
MITP: Mother Infant Transaction Programme
M-MITP: Modified mother infant transaction programme
NBAS: Neonatal Behavioral Assessment Scale
NICU: Neonatal Intensive Care Unit
NIDCAP: Newborn Individualised Developmental & Assessment Programme
NSTEP-P: Nursing Systems Towards Effective Parenting-Preterm
PBIP: Parent-Baby Interaction Programme
PI: Physiotherapy Intervention
PICOS: Population, Intervention, Comparator, Outcome, Study design
PRISMA: Preferred Reporting Items for Systematic Reviews and Meta Analyses
RCTs: Randomised Controlled Trials
RR: Relative Risk
SM: State Modulation
SM-NSTEP-P: State Modulation-Nursing System Towards Effective Parenting – Preterm
SPEEDI: Support Play Exploration and Early Development Intervention
SRs: Systematic Reviews
TH: Traditional Holding
VIBeS PLUS: Victorian Infant Brain Studies Program
